# Correlation study of NF-κB, IER3, and Recurrence of Ovarian Endometrioid Cysts

**DOI:** 10.1007/s43032-024-01722-5

**Published:** 2024-10-08

**Authors:** Xiang Fan, Ni Yang, Gu Huang, Yishan Dong, Pengfeng Zhu

**Affiliations:** 1 Department of Gynecology, Changzhou Maternity and Child Health Care Hospital, Changzhou Medical Center, Nanjing Medical University, Changzhou, 213000 China; 2 Department of Gynecology, Bazhong Central Hospital, Bazhong, 636600 China

**Keywords:** Ovarian endometrioid cyst, Recurrence, NF-κB, IER3

## Abstract

The study aimed to investigate the expression of nuclear actor-k-gene binding(NF-κB) and immediate early response 3(IER3) in ovarian endometrioid cysts and its correlation with the recurrence of the ovarian endometrioid cyst. From January 2018 to March 2019, a total of 88 patients who underwent laparoscopic ovarian cyst excision due to ovarian endometrioid cyst in Changzhou Maternity and Child Health Care Hospital were selected. Clinical data of the patients were collected. The patient's Revised American Fertility Society (R-AFS) score, least function(LF) score, and endometriosis fertility index (EFI) were calculated. Immunohistochemistry was performed to detect the expression of IER3 and NF-κB. The receiver-operating characteristic (ROC) curve was used to evaluate the predictive value of IER3 and NF-κB expression on postoperative recurrence. Cox regression was fitted to analyze the influencing factors of ovarian endometrioid cyst recurrence. The expression of NF-κB was positively correlated with IER3 (*P* < 0.001). ROC curve showed that NF-κB combined with IER3 had higher predictive value for disease recurrence. Multivariate Cox regression showed that the IER3 expression intensity > 4.5 (HR = 3.418,95%CI: 1.227 ~ 9.523, *P* = 0.019) and the NF-κB expression intensity > 4.5 (HR = 5.491,95%CI: 1.600 ~ 18.838, *P* = 0.007) were independent risk factors for recurrence, and EFI score (HR = 0.791,95%CI: 0.637 ~ 0.983, *P* = 0.035) was a protective factor for recurrence. Our results suggested that EFI score is a protective factor for recurrence. The expression levels of NF-κB and IER3 > 4.5 are correlated with the recurrence of ovarian endometrioid cysts and independent risk factors for recurrence.

## Introduction

Endometriosis (EMs) plays an important role in gynecological diseases. The characteristics of recurrence, infertility, and pain cause a serious burden on women's bodies and minds. There is still a lack of reliable biomarkers for early diagnosis of recurrent endometriosis. The ovarian endometrioid cyst is the most common pathological type in EMs. So far, its treatment methods are mainly surgery and drug therapy. Although there are many emerging surgical methods nowadays, such as ultrasound-guided cyst puncture, cyst puncture combined with sclerotherapy, etc. Although the trauma is less, it is limited to a single lesion and requires no adhesion or damage to the lesion. The first choice for diagnosis and treatment of ovarian endometrioid cyst is still laparoscopic endometrioid cyst excision, but the recurrence rate is high. Finding effective prognostic markers to screen patients with possible recurrence can greatly improve the quality of life of patients and reduce the recurrence rate after surgery.

Nf-κB is a dimeric transcription factor that promotes the expression of more than 150 genes involved in cellular processes, including immune responses and inflammatory processes [1]. The NF-κB pathway has been considered a classical proinflammatory signaling pathway, mainly based on the role of NF-κB in the expression of proinflammatory genes, including inflammatory mediators, cytokines, adhesion molecules, apoptosis inhibitors, and chemokines. The pleiotropic result of its activated nuclear factor is caused by the combinatorial effects of the five subunits forming homologous and heterodimeric NF-κB complexes [2]. Nf-κB can be activated by oxidants and cytokines, and then cause endometriosis. Subsequently, NF-κB plays an important role in the maintenance of oxidative stress [3]. Song et al. [4] found that down-regulation of NF-κB could reduce oxidative stress and inflammation, thereby alleviating nephropathy induced by doxorubicin. Nf-κB plays an important role in the development and progression of cancer and is a new target for the diagnosis or treatment of tumors [5].

IER3 is a stress-inducing gene that can be rapidly regulated by a variety of factors, including transcription factors, inflammatory cytokines, viral infections, chemical carcinogens, growth factors, and hormones, et al. [6]. IER3 plays a key role in the development of cancer and can be used as a prognostic predictor for some tumors, such as bladder cancer, liver cancer, and ovarian cancer [7]. IER3 has been established as a target gene of NF-κB in response to stress, and studies have shown that IER3 can be induced and activated through the NF-κB pathway [8].

Endometriosis is an estrogen-dependent inflammatory disease, as well as a disease associated with high levels of chronic oxidative stress [9], and has similar characteristics to tumors in many aspects, such as invasiveness, adhesion, and metastatic potential. As mentioned above, NF-κB plays an important role in inflammation, oxidative stress, and tumor development. IER3 is a target gene of NF-κB and a prognostic predictor of a variety of tumors. In recent years, NF-κB and IER3 have been widely studied in the field of tumor prevention and treatment. Based on the correlation between endometriosis and inflammation and oxidative stress, and its similar characteristics to tumors, this study intends to detect the expression of NF-κB and IER3 in recurrent and non-recurrent ovarian endometrioid cyst tissues by immunohistochemical method, and investigate the correlation between NF-κB and IER3 and the recurrence of the ovarian endometrioid cyst.

## Materials and Methods

### Experimental Materials and Methods

#### Specimen Source and Clinical Data Collection

**Fig. 1:**
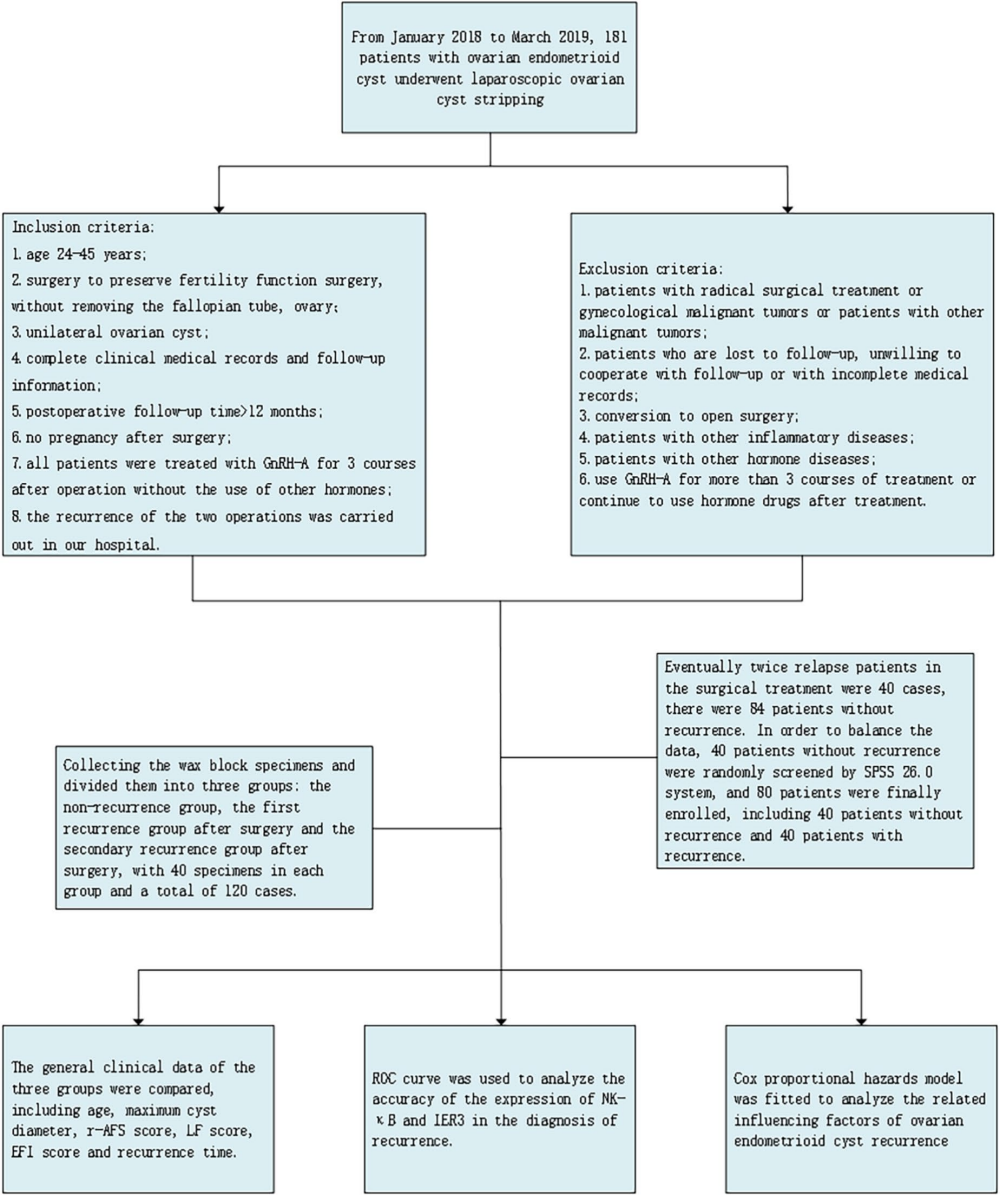
The flow chart designed in this study

A total of 181 patients with ovarian endometrioid cysts who underwent laparoscopic ovarian cyst excision in the Department of Gynecology, Changzhou Maternal and Child Health Hospital from January 2018 to March 2019 were selected. All patients were performed by the same surgeon and her team. All patients were confirmed to be ovarian endometrioid cysts by pathological results after surgery. The end time of follow-up was June 30, 2024, or the second surgical visit date for patients with recurrence. Follow-up methods include telephone and database. General clinical data were collected, including age, ovarian cyst diameter, R-AFS score, LF score, EFI score, and recurrence time. Informed consent was obtained from the patients before obtaining the specimens and medical records, which were approved by the Ethics Committee of Changzhou Maternal and Child Health Hospital affiliated with Nanjing Medical University. The flow chart designed in this study is shown in Fig. 1.

#### Inclusion and Exclusion Criteria

Inclusion criteria: 1. age 24–45 years; 2. surgery to preserve fertility function surgery, without removing the fallopian tube, ovary; 3. Unilateral ovarian cyst; 4. Complete clinical medical records and follow-up information; 5. Postoperative follow-up time > 12 months; 6. No pregnancy after surgery; 7. All patients were treated with GnRH-a for 3 courses after operation without the use of other hormones; 8. The recurrence of the two operations was carried out in our hospital.

Exclusion criteria: 1. patients with radical surgical treatment or gynecological malignant tumors or patients with other malignant tumors; 2. patients who are lost to follow-up, unwilling to cooperate with follow-up or with incomplete medical records; 3. conversion to open surgery; 4. patients with other inflammatory diseases; 5. patients with other hormone diseases; 6. use GnRH-a for more than 3 courses of treatment or continue to use hormone drugs after treatment.

After screening, 20 patients with postoperative pregnancy, and 13 patients with a bilateral ovarian cyst, medical records are not complete, and not willing to accept the follow-up of patients with 12 cases, more than three courses of treatment with GnRH-a or continue to use hormonal drugs after treatment of 30 cases and 7 patients with other diseases, these cases were excluded. Eventually, twice relapse patients in the surgical treatment were 55 cases, there were 44 patients without recurrence. In order to balance the data, 55 twice relapse patients were randomly screened by SPSS 26.0 system, and 88 patients were finally enrolled, including 44 patients without recurrence and 44 patients with recurrence. Two operations were performed in our hospital for patients with recurrence, including the first operation after recurrence and the second operation after recurrence. We collected the wax block specimens archived in the pathology department and divided them into three groups: the non-recurrence group, the first recurrence group after surgery and the secondary recurrence group after surgery, with 44 specimens in each group and a total of 132 cases.

### Immunohistochemistry Was Used to Detect the Expression of NF-κB and IER3 in Each Tissue

#### IHC Procedure

The sections were dewaxed in xylene and hydrated in concentration gradient alcohol. The sections were heated with repair solution in a pressure cooker at 140℃ for 2.5 min and treated with H2O2 solution at indoor temperature for 10 min to complete antigen repair. Add primary antibody according to kit instructions and incubate overnight at 4℃. The next day, the corresponding secondary antibody was added and allowed to stand at indoor temperature for 20 min. Then the colorant was added by drop. After dehydration in concentration gradient alcohol, the tablets were transparent with xylene for 2 min, and the slide was sealed after drying.

#### Interpretation of Immunohistochemical Results

The positive staining of NF-κB was mainly located in the cytoplasm and nucleus, and the positive staining of IER3 was mainly located in the cell membrane and cytoplasm, and the positive staining was brown. The sections were observed under a high-power microscope, and high-definition images were collected. The degree of staining was evaluated by two pathology experts who don't know the clinical information of the patients. The positive staining intensity and the number of cells in each field were counted and averaged to obtain the final staining fraction. NF-κB and IER3 staining scores were determined by staining intensity (0–3 grade: level 0 was negative, level 1 was weak positive, level 2 was medium positive, level 3 was strong positive) and percentage of positive cells (0–4 grade: level 0 was 0%, level 1 was 1%-25%, level 2 was 26%-50%, level 3 was > 50%), divided into different fractions (0, 1, 2, 3, 4, 5, 6). 0–1 was defined as negative expression, 2–3 as weak positive expression, 4–5 as moderate positive expression, and 6 as strong positive expression.

### Statistical Analysis

Statistical software SPSS 26.0 was used for data analysis. Measurement data were expressed as Mean ± SD, and enumeration data were expressed as percentage or rate. Kruskal–Wallis test was used for multiple comparisons between groups. Spearman rank correlation test was used to analyze the correlation between rank data and count data. Pearson rank correlation test was used to analyze the count data. Cox proportional hazards model was fitted to analyze the related influencing factors of ovarian endometrioid cyst recurrence, and *P* < 0.05 was considered statistically significant.

## Result

A total of 132 specimens were included in this study, including 44 cases in the non-recurrence group, 44 cases in the first recurrence after surgery group(first recurrence group), and 44 cases in the secondary recurrence after surgery group(second recurrence group). The specific clinical data are shown in Table 1.

**Table 1:** General clinical data of the patients

	Groups			*H*	*P*
	Non-recurrence group	First recurrence group	Second recurrence group		
Number	44	44	44		
Age	34.05 ± 5.14	35.09 ± 5.49	-	1.692	0.193
Maximum cyst diameter (cm)	5.14 ± 1.37	5.07 ± 1.04	4.86 ± 0.93	1.045	0.593
r-AFS score	31.95 ± 8.469	35.18 ± 12.22	39.73 ± 11.87*	12.791	0.002
LF score	4.93 ± 2.07	4.27 ± 1.92	4.73 ± 2.08	2.722	0.256
EFI score	6.68 ± 1.39	3.91 ± 1.58*	3.45 ± 1.44*	63.480	< 0.001

Compared with the non-recurrence group, ^*^P < 0.05; Compared with the first recurrence group, ^#^*P* < 0.05

**Fig. 2:**
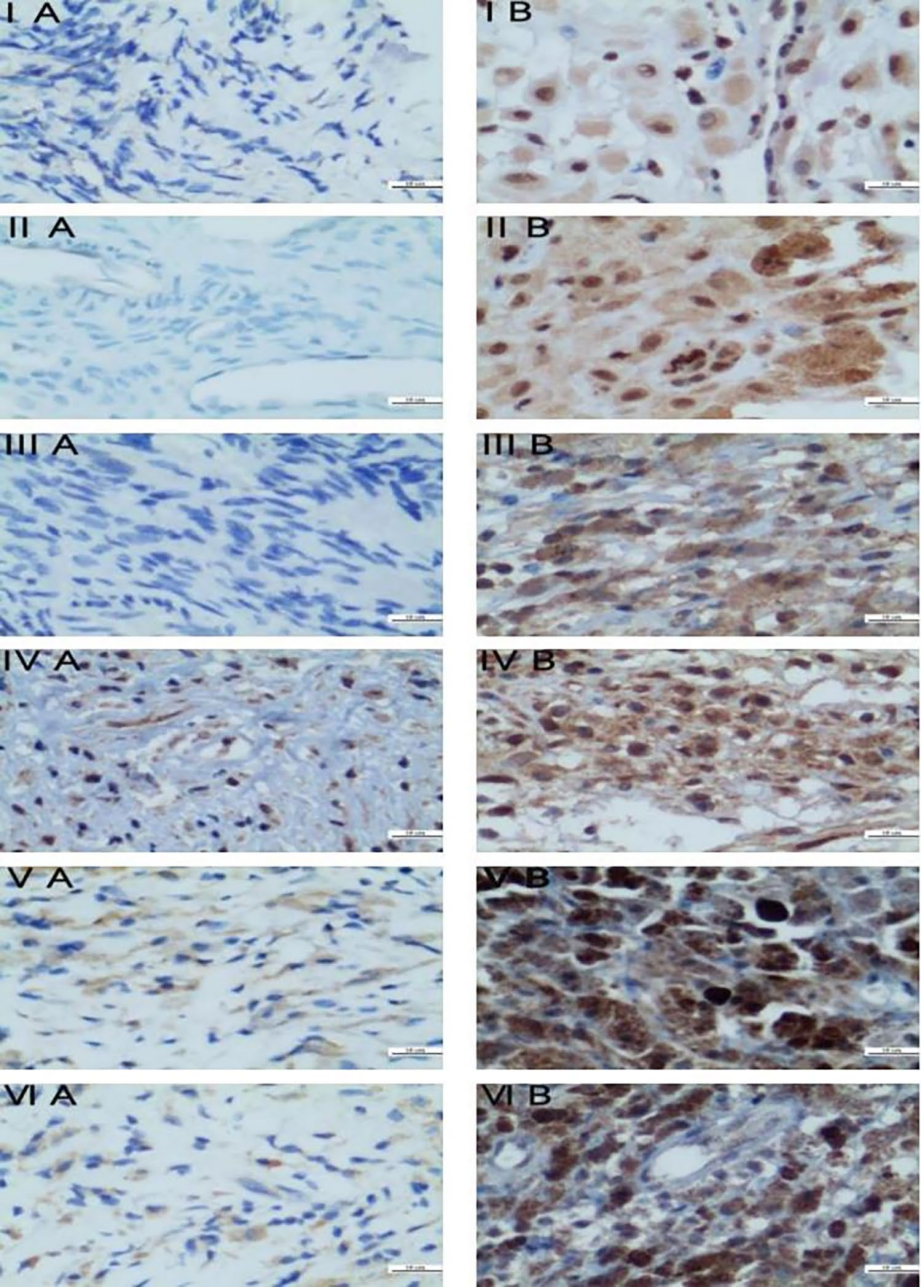
Expression of NF-κB and IER3 in each group (SP, × 400)

NF-κB and IER3 were positively expressed in the three groups (Fig. 2). There were 44 cases in the non-recurrence group, 44 cases in the first recurrence group, and 44 cases in the secondary recurrence group. The expression intensity of NF-κB and IER3 in the first recurrence group was significantly different from that in the non-recurrence group (*P* < 0.05). And the expression intensity of NF-κB and IER3 in the second recurrence group was significantly different from that in the first recurrence group (*P* < 0.05), as shown in Table 2.

**Table 2:** Expression of NF-κB and IER3 in each group

	Number	NF-κB expression				IER3 expression			
		**-**	** + **	** + + **	** + + +**	**-**	** + **	** + + **	** + + + **
Non-recurrence group	44	5	26	11	2	4	28	9	3
First recurrence group	44	1	5	17	21*	2	6	10	26*
Second recurrence group	44	8	24	10	2^#^	3	20	18	3^#^
*H*		45.242				36.240			
*P*		< 0.001				< 0.001			

Compared with the non-recurrence group, *P < 0.05; Compared with the first recurrence group, ^#^*P* < 0.05

Spearman analysis showed that the positive degree of IER3 was positively correlated with the positive degree of NF-κB in the three groups (Table 3). The correlation between the expression of NF-κB and IER3 and R-AFS score, LF score and EFI score in each group is shown in Table 4.

**Table 3:** Correlation between IER3 and NF-κB expression in non-recurrence group and first recurrence group (Spearman)

	*r*_*s*_	*P*
Non-recurrence group	0.858	< 0.001
First recurrence group	0.731	< 0.001
Second recurrence group	0.718	< 0.001

**Table 4:** Relationship between the expression of NF-κB and IER3 in each group and general clinical data (Pearson)

	r-AFS score		LF score		EFI score	
	*r*	*P*	*r*	*P*	*r*	*P*
Non-recurrence group						
NF-κB	0.361	0.016	-0.352	0.019	-0.361	0.016
IER3	0.593	< 0.001	-0.347	0.021	-0.299	0.049
First recurrence group						
NF-κB	0.444	0.003	-0.399	0.007	-0.584	< 0.001
IER3	0.441	0.003	-0.357	0.017	-0.592	< 0.001
Second recurrence group						
NF-κB	0.424	0.004	-0.505	< 0.001	-0.327	0.030
IER3	0.348	0.020	-0.614	< 0.001	-0.418	0.005

**Fig. 3:**
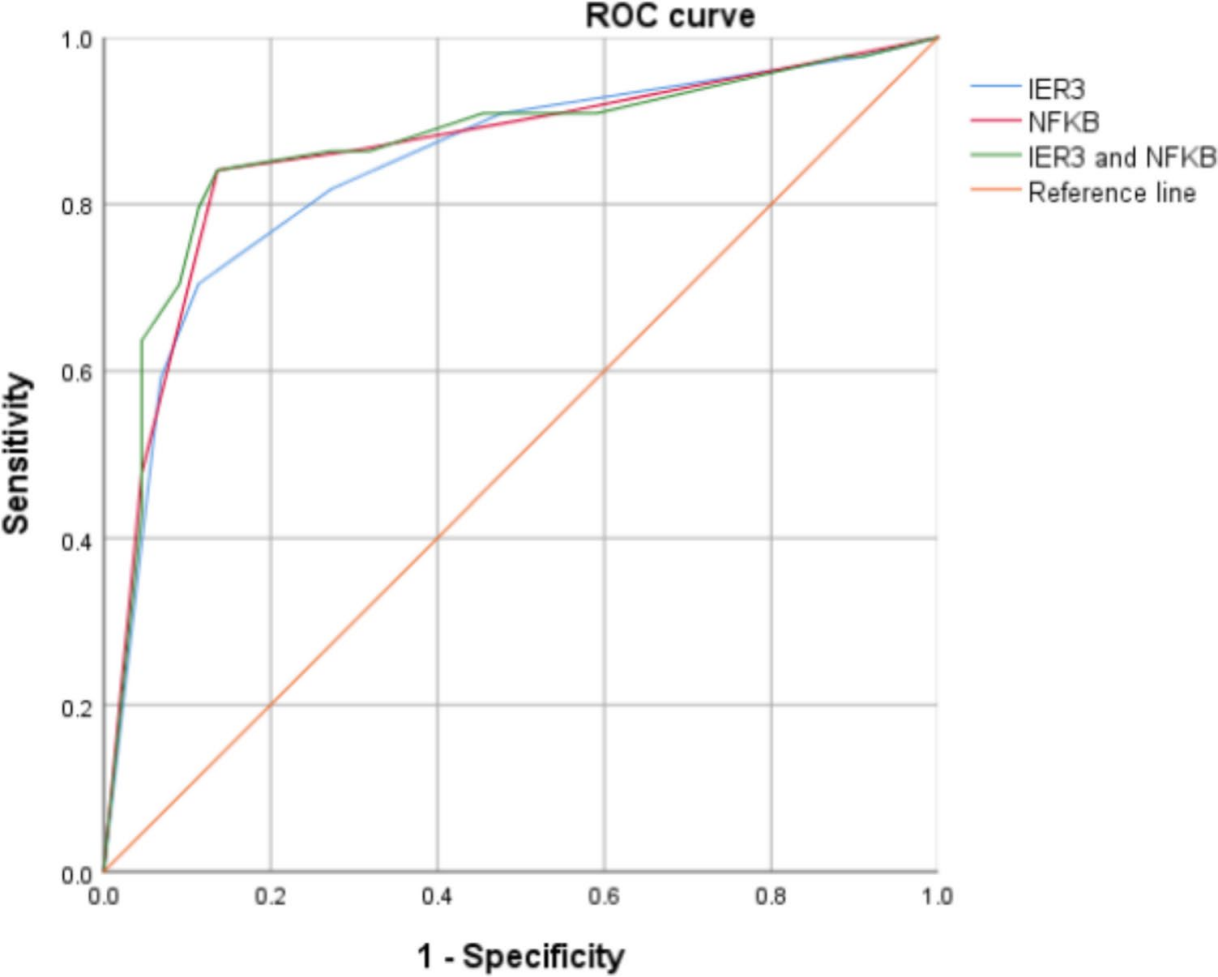
ROC curves of NF-κB and IER3 after the primary operation

According to the receiver operating characteristic curve (ROC), the cut-off value of NF-κB expression score after the initial operation was 4.5, the area under the curve was 0.862, *P* < 0.05, 95% confidence interval was 0.779 to 0.945, and Youden index was 0.705. The cut-off value of the IER3 expression score was 4.5, the area under the curve was 0.845, *P* < 0.05, 95% confidence interval was 0.759 to 0.930, and the Youden index was 0.591. The combination of the two has the highest diagnostic accuracy (Fig. 3 and Table 5).

**Table 5:** Combined expression of IER3 and NF-κB predicts ovarian endometrioid cyst recurrence

	NF-κB	IER3	Combined expression
Sensitivity(%)	0.841	0.705	0.841
Specificity(%)	0.864	0.886	0.864
AUC	0.862	0.845	0.867
95% CI	0.779 ~ 0.945	0.759 ~ 0.930	0.784 ~ 0.950

IER3 and NF-κB were correlated with R-AFS score, LF score and EFI score, the above five variables were included in multivariate COX survival analysis by stepwise regression method. The final model included four variables, IER3, NF-κB, r-AFS score and EFI score. IER3 expression > 4.5 (HR = 3.418,95%CI: 1.227 ~ 9.523, *P* = 0.019), NF-κB expression > 4.5 (HR = 5.491,95%CI: 1.600 ~ 18.838, *P* = 0.007) was an independent risk factor for postoperative recurrence, EFI score (HR = 0.791,95%CI: 0.637 ~ 0.983, *P* = 0.035) was a protective factor for postoperative recurrence, as shown in Table 6.

**Table 6:** Multivariate Cox survival regression analysis

	Univariate			Multivariate		
	*HR*	*95% CI*	*P*	*HR*	*95% CI*	*P*
IER3(> 4.5/ < 4.5)	15.982	7.656 ~ 33.360	< 0.001	3.418	1.227 ~ 9.523	0.019
NF-κB(> 4.5/ < 4.5)	21.684	9.320 ~ 50.449	< 0.001	5.491	1.600 ~ 18.838	0.007
r-AFS score	1.026	1.001 ~ 1.052	0.043	0.989	0.957 ~ 1.022	0.520
LF score	0.898	0.777 ~ 1.036	0.140	-	-	-
EFI score	0.525	0.441 ~ 0.624	< 0.001	0.791	0.637 ~ 0.983	0.035

## Discussion

In recent years, the incidence of endometriosis is about 10–15% and has a trend of increasing year by year [10]. More and more evidence has confirmed the multifactorial nature of endometriosis, which is the result of the combined effects of anatomical, immune, reactive, hormonal, genetic, epigenetic, and environmental factors in affected women ADDIN NE.Ref.{1511EB04-57F2-4041-B93A-42138CBE56EA}[11]. Guideline for the diagnosis and treatment of endometriosis recommend that medical treatment be preferred in patients without infertility and those with adnexal masses < 4 cm in diameter. Surgery may be considered in patients with infertility or adnexal mass ≥ 4 cm in diameter, and in patients who do not respond to medical therapy ADDIN NE.Ref.{22DB0C3E-24C6-4B06-835D-3D710DD2EF08}[12]. Currently, laparoscopic surgery is still the standard treatment for ovarian endometrioid cysts ADDIN NE.Ref.{090537C9-556D-48858685-9201FCC1B28E}[13, 14]. The recurrence rate of ovarian endometrioid cysts in patients without appropriate postoperative treatment can be as high as 40% within 2 years ADDIN NE.Ref.{406576F0-A3E1-48B6-8389-F506F85C0E03}[15]. Medical treatment is recommended for patients who do not desire fertility after surgery. First-line drugs include non-steroid anti-inflammatory drug (NSAID), oral contraceptives and high-potency progesterone, and second-line drugs include GnRH-a and levonorgestrel intrauterine system (LNG-IUS) ADDIN NE.Ref.{29,400,857 7126-464B-BC8C-AF92734ED9D9}[12]. At present, GnRH-a is still the "gold standard" for drug treatment of endoheterosis ADDIN NE.Ref.{156D05DA-6969-4CDD-9557-AADDAF504D0A}[12]. Pharmacologic doses of GnRH agonists administered for six months have been found to produce amenorrhea and anovulation in numerous controlled, randomized clinical studies ADDIN NE.Ref.{432D0E97-0166-4E7E-B761-BB710E0CD3F7}[16]. Therefore, the patients included in this study were treated with GnRH-a for only 3 times after surgery, and the patients were followed up closely after treatment. Even for patients receiving appropriate postoperative treatment, the recurrence rate is still about 10% ADDIN NE.Ref.{08D175DC-0607-4A12-9CB3-523E8064C28F}[17]. For such patients, if they have no fertility requirements, surgery or ultrasound-guided puncture can be performed. GnRH-a therapy is recommended to be given after surgery, and then other drugs may be used for long-term maintenance therapy ADDIN NE.Ref.{6224759E-7EF4-421B-8AE9-A32970281C9C}[12]. Due to such a high recurrence rate and the lack of effective biological markers, many scholars have started to study the related cell, protein and gene biomarkers in urine, blood, cervical mucus and other body fluids from a non-invasive perspective, but the results are still not ideal.

The NF -κB pathway is one of the major markers of inflammation, which can regulate cell proliferation, apoptosis and inflammatory processes [18]. NF-κB regulates various proinflammatory such as Tumor necrosis factor(TNF-α), Interleukin(IL-1, IL-6, IL-12), chemokines(CXCL1, CXCL2), recombinant human chemokine-5, adhesion molecules(vascular cell adhesion factor-1) and so on, involved in the activation and recruitment of inflammatory regulatory cells. The NF-κB pathway is down-regulated in normal endometrium [19], but its expression is increased in all stages of endometriosis [20]. Previous studies have demonstrated that women with endometriosis have increased NF-κB expression that regulates the expression of aberrant cytokines through autocrine self-amplifying cycles of cytokine release and NF-κB activation. These lead to amplification and maintenance of the proinflammatory local environment, promoting the survival and growth of endometrial cells in endometriosis patients and reducing the clearance of retrogradely transported endometrial fragments, which promotes the development of endometriosis [21, 22]. Therefore, given the high recurrence rate of endometriosis, we hypothesized that high expression of NF-κB may lead to the formation and maintenance of proinflammatory local environment that allows the growth and invasion of ectopic endometrial cells, which promotes the recurrence of endometriosis.

In our study, the expression intensity of NF-κB in the first recurrence group was higher than that in the non-recurrence group. As mentioned above, NF-κB plays an important role in the occurrence and development of ovarian endometrioid cysts. Patients with recurrent endometrioid cysts may be more prone to recurrent ovarian endometrioid cysts due to stronger expression of NF-κB and more severe inflammatory reactions in vivo. Nf-κB can regulate the expression of genes and play a key role in the development and progression of cancer, especially in inflammatory tumors, such as the proliferation, migration and apoptosis of cancer cells, and is related to the occurrence of lung cancer [23], nasopharyngeal carcinoma [23], breast cancer [5], liver cancer [24] and other tumors. In recent years, NF-κB has also been studied as a new target for the diagnosis or treatment of tumors, such as endocrine therapy of breast cancer [5]. In the study of the tumor and inflammatory diseases, scholars have found that EMs seems to fit another disease – endometriosis very well. EMs has many characteristics similar to malignant cells, such as invasion and proliferation of ectopic cells. In this study, the high expression of NF-κB in the recurrence group suggests that the recurrence group is more prone to adhesion and invasion. Nf-κB may also be used as a target for the treatment of ovarian endometrioid cysts, reducing the inflammatory response of patients with ovarian endometrioid cysts by reducing the expression of NF-κB, to achieve the purpose of controlling the recurrence.

Gonzalez-ramos et al. [22] found that the NF-κB pathway is related to the occurrence of endometriosis in the early development stage. In this study, the expression intensity of NF-κB in the first recurrence group and the recurrence group was significantly higher than that in the non-recurrence group, and NF-κB promoted the occurrence of ectopic lesions. One of the NF-κB subunits of Rel family proteins is p65, which assembles as a dimer in the cytoplasm. In the inactive state, NF-κB binds to its inhibitor IκB, forming the NF-κb-IκB complex. After activation, NF-κB translocates to the nucleus, where IκB is phosphorylated in response to different stimuli. IL-6 and IL-8 in endometriosis can thus be activated. Inhibition of the action of the complex can reduce the maintenance and development of Ems [25], so patients with higher NF-κB expression are more likely to develop endometriosis. The expression of NF-κB was stronger in the patients with postoperative recurrence in this study, which can be speculated that the higher the expression of NF-κB, the stronger the inflammation, and the easier the recurrence of the ovarian endometrioid cyst.

IER3 plays a key role in the regulation of cell proliferation and apoptosis, and its overexpression can inhibit or enhance cell apoptosis according to the nature of stress [26]. Positive expression of IER3 in ovarian cancer [7] and pancreatic cancer [27] is associated with a good prognosis, while increased expression of IER3 in other diseases has a poor survival rate, such as acute myeloid leukemia, bladder cancer, liver cancer, breast cancer, Sezary syndrome and colorectal cancer [6], this may be related to the excess IER3 mediating cancer cell survival. IER3 has been widely reported in cancer, but rarely in EMs with oxidative stress and inflammatory diseases. Given the great advances in diagnostic imaging (such as transvaginal ultrasound and nuclear magnetic resonance), the diagnosis of endometrioid cysts tends to be simplified and become a structured process based on a combination of patient interviews, clinical examination and imaging. The diagnosis of endometriosis is not difficult, but recurrence is very troublesome [28]. Endometriosis is deeply affected by oxidative stress. Samimi et al. [29] found that the concentrations of glutathione peroxidase and catalase were higher in EMs ectopic cells, and in EMs follicular fluid, Higher concentrations of malondialdehyde (MDA), NO and reactive oxygen species (ROS) were observed. As a stress-inducer gene, IER3 was involved in oxidative stress and inflammatory reactions. Reactive oxygen species (ROS) can also activate the NF-κB pathway, promote oxidative stress-mediated proinflammatory signals, and promote the development of endometriosis [30]. The IER3 promoter has been shown to contain consistent sequences of several transcription factors, including NF-κB, p53, cMyc, VDRE, Sp1, p300, and Sox [31]. Studies have shown that IER3 plays a regulatory role in apoptosis, inflammation, immune system regulation, tumorigenesis and other cellular functions mainly through NF-κB, PI3K/Akt and MAPK/ERK pathways [6]. Therefore, we hypothesized that NF-κB promotes the development of endometriosis by targeting IER3 expression induced by inflammation and oxidative stress. The results of this study were consistent with that of IER3 expression in almost all ovarian endometrioid cysts, and the expression intensity of IER3 in the recurrence group was higher than that in the non-recurrence group, and the difference was statistically significant (*P* < 0.05), indicating that the patients with recurrent ovarian endometrioid cysts had strong oxidative stress and inflammatory response in the body. The high expression of IER3 provides a good basis for the recurrence of the ovarian endometrioid cyst.

In this study, the expression of NF-κB and IER3 was highly expressed in patients with recurrent ovarian endometrioid cysts. The area under the ROC curve of NF-κB and IER3 expression score in the tissues of patients with ovarian endometrioid cysts in the primary operation was 0.867. NF-κB and IER3 have a high accuracy in predicting the recurrence of ovarian endometrioid cysts, which may be related to the disease recurrence by enhancing the inflammatory and oxidative stress response in vivo. Multivariate Cox survival regression showed that the expression of IER3 > 4.5 (HR = 3.418,95%CI: 1.227 ~ 9.523, *P* = 0.019), NF-κB > 4.5 (HR = 5.491,95%CI: 1.600 ~ 18.838, *P* = 0.007) was an independent risk factor for postoperative recurrence, and EFI score (HR = 0.791,95%CI: 0.637 ~ 0.983, *P* = 0.035) was a protective factor for postoperative recurrence. The expression levels of NF-κB and IER3 in the two recurrence group was significantly higher than those in the non-recurrence group, which also suggested that the level of oxidative stress in patients with recurrent ovarian endometrioid cyst was higher. Therefore, drugs targeting NF-κB and IER3 may inhibit the recurrence of ovarian endometrioid cysts.

The high expression of NF-κB and IER3 suggests that the risk of ovarian endometrioid cyst recurrence may be higher. For patients who are more prone to recurrence, more systematic treatment should be taken to avoid recurrence after surgery. Studies have shown that an NF-κB inhibitor, DHMEQ, inhibited the migration and invasion of human endometriosis stromal cells, and DHMEQ is particularly effective in suppressing disease models by intraperitoneal administration [32]. Therefore, we can further study the effects of NFKB inhibitors and IER3 inhibitors on endometriosis stromal cells, so as to further verify the inhibitory effect of these inhibitors on endometriosis through animal models, with a view to developing drugs that can more effectively inhibit the occurrence and development of endometriosis. Moreover, patients who plan to become pregnant should be encouraged to become pregnant as early as possible, and individualized precise medication should be realized to reduce patients' pain. Regrettably, the sample size of this study is relatively small, the data results are not representative, and other types of EMs cases (such as deep infiltrating type and superficial peritoneal EMs, etc.) are not included. Therefore, the sample size should be expanded and the types of cases should be enriched in subsequent research to get more convincing conclusions.

## Data Availability

The data that support the findings of this study are available on request from the corresponding author. The data are not publicly available due to privacy or ethical restrictions.
